# £150,000 Required

**Published:** 1919-03-29

**Authors:** 


### THE NEW NURSES' HOME AT ST. BARTHOLOMEW'S.

### £150,000 Required.

MR. MCADAM ECCLES, who has been chosen by his colleagues for the onerous post of Honorary Secretary of the St. Bartholomew's Hospital Nurses' Home Fund, is not taking his responsibilities lightly. Prior to laying the outline of a scheme for the Home before the Board of Governors, which met on March 21 and unanimously approved it, Mr. Eccles journeyed near and far to get ideas, visiting the leading Nurses' Homes in the Midlands and the North as far as Newcastle-on-Tyne, besides the newest buildings of the kind in London. The architect who is to assimilate and reproduce whatever has been found to promote the welfare of nurses is Mr. E. T. Mathews, architect of the hospital, and he will have the advantage of Mr. Edwin T. Hall's advice in consultation.

The building has been planned to take 400 nurses, besides at least 100 ward maids and domestics. This number is nearly 100 more than the present staff, but they are wisely building for the future. The necessary site will be obtained by clearing the ground in Little Britain of the heterogeneous collection of unsatisfactory buildings at present used for sleeping purposes by the nurses' staff. Alas, this clearance cannot be effected without at the same time destroying what may be deemed one of the most finely-proportioned rooms in London, the old hall of Christ's College, now used as a nurses' sitting-room. But this sacrifice, it seems, cannot be avoided.

The Home will be fitted in between the gigantic Post Office structure and the South Block of the Hospital. Constructed entirely of ferro-concrete, architectural adornments will not be a feature, and perhaps, considering the Home's ancient neighbours, it is well advised to aim modestly at space, light, air and convenience instead. Every inmate of the Home will have her own bedroom. The various floors are to be arranged as complete units, so that in the event of an infectious case occurring the whole floor can be shut off. To each six or seven bedrooms there will be a bathroom, and the question of supplying each bedroom with hot and cold water turned on is under discussion. There will be shampoo-rooms, tea-rooms, and work-rooms, with conveniences for doing a little private laundry work. The night nurses' quarters will be separate and fitted with double doors to exclude noise. The community rooms will include lounge, music-room and visitors' room, and the lecture theatre will be designed with a view to use for the performance of plays and for dancing. The nurses of the private nurses' staff will be housed in a self-contained maisonette.

A somewhat novel feature, copied from a similar department at the Newcastle-on-Tyne Royal Victoria Infirmary, will be a Post Office for the receipt and despatch of letters and parcels and the transaction of ordinary business.

The estimated cost is £150,000, by no means an excessive computation, it would seem, at present prices. Many of the Governors are making themselves responsible for raising £350 each, and the amount already promised is gratifying.

